# No differences in native T1 of the renal cortex between Fabry disease patients and healthy subjects in cardiac-dedicated native T1 maps

**DOI:** 10.1016/j.jocmr.2024.101104

**Published:** 2024-09-25

**Authors:** Anna Damlin, Felix Kjellberg, Raquel Themudo, Kelvin Chow, Henrik Engblom, Mikael Oscarson, Jannike Nickander

**Affiliations:** a Department of Clinical Physiology, Karolinska University Hospital, Stockholm, Sweden; b Department of Cardiology, Karolinska University Hospital, Stockholm, Sweden; c Department of Clinical Science, Intervention and Technology at Karolinska Institutet, Division of Medical Imaging and Technology, Stockholm, Sweden, Stockholm, Sweden; d Cardiovascular MR R&D Siemens Medical Solutions Inc. Chicago, USA; e Department of Endocrinology, Centre for Inherited Metabolic Diseases, Karolinska University Hospital, Stockholm, Sweden; f Department of Molecular Medicine and Surgery, Karolinska Institutet, Stockholm, Sweden; g Department of Radiology, Karolinska University Hospital in Huddinge, Stockholm, Sweden

**Keywords:** Fabry disease, Lysosomal storage diseases, Magnetic resonance imaging, Kidney failure

## Abstract

**Background:**

Fabry disease (FD) is an X-linked inherited lysosomal storage disease that is caused by deficient activity of the enzyme alpha-galactosidase A. Cardiovascular magnetic resonance (CMR) imaging can detect cardiac sphingolipid accumulation using native T1 mapping. The kidneys are often visible in cardiac CMR native T1 maps; however, it is currently unknown if the maps can be used to detect sphingolipid accumulation in the kidneys of FD patients. Therefore, the aim of this study was to evaluate if cardiac-dedicated native T1 maps can be used to detect sphingolipid accumulation in the kidneys.

**Methods:**

FD patients (n = 18, 41 ± 10 years, 44% (8/18) male) and healthy subjects (n = 38, 41 ± 16 years, 47% (18/38) male) were retrospectively enrolled. Native T1 maps were acquired at 1.5T using modified Look-Locker inversion recovery research sequences. Native T1 values were measured by manually delineating regions of interest (ROI) in the renal cortex, renal medulla, heart, spleen, blood, and liver. Endo- and epicardial borders were delineated in the myocardium and averaged across all slices. Blood ROIs were placed in the left ventricular blood pool in the midventricular slice.

**Results:**

There were no differences in native T1 between the FD patients and the healthy subjects in the renal cortex (1034 ± 88 ms vs 1056 ± 59 ms, p = 0.29), blood (1614 ± 111 ms vs 1576 ± 100 ms, p = 0.22), spleen (1143 ± 45 ms vs 1132 ± 70 ms, p = 0.54), or liver (568 ± 49 ms vs 557 ± 47 ms, p = 0.41). Native myocardial T1 was lower in FD patients compared to healthy subjects (951 ± 79 vs 1006 ± 38, p<0.01), and higher in the renal medulla (1635 ± 144 vs 1514 ± 81, p<0.01).

**Conclusion:**

Compared to healthy subjects, patients with FD and cardiac involvement showed no differences in native T1 of the renal cortex. FD patients had higher native T1 in the renal medulla, which is not totally explained by differences in blood native T1 but may reflect a hyperfiltration state in the development of renal failure. The findings suggest that sphingolipid accumulation in the renal cortex in FD patients could not be detected with cardiac-dedicated research native T1 maps.

## Graphical abstract

**Figure:**
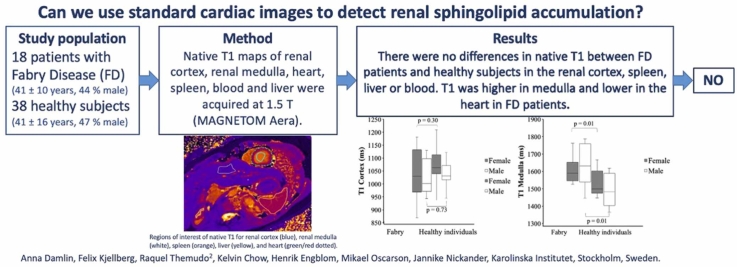

## 1 Background

Fabry disease (FD) is an X-linked inherited disorder caused by deficient activity of the lysosomal enzyme alpha-galactosidase A [1]. The deficiency causes accumulation of the sphingolipid globotriaosylceramide in several different types of tissues [2] leading to, for instance, progressive renal failure, neuropathic pain, and heart failure due to left ventricular hypertrophy, as well as other cardiac abnormalities such as conduction defects, coronary artery disease, valvular involvement, and hypertension [1], [2], [3], [4]. Renal involvement in patients with FD is due to the accumulation of sphingolipids in several different types of renal cell types, which leads to glomerular disorders such as albuminuria, early hyperfiltration, and progressive reduction of renal function [5].

Cardiovascular magnetic resonance (CMR) imaging is an excellent method for detecting and assessing cardiac involvement of FD, even in the early stages [6], [7], [8], [9], [10]. FD is characterized by sphingolipid accumulation, which can be detected by CMR using native T1 mapping, as native T1 is decreased compared to healthy individuals [7], [8]. Currently, there is no clear consensus on the best approach to assess renal involvement in FD [11], [12]; however, light microscopy of renal biopsies is widely used [13], [14]. Magnetic resonance imaging can highlight renal abnormalities where loss of corticomedullary differentiation is associated with increased T1 relaxation times in the cortex [15]. It is common for FD patients to undergo CMR, and the kidneys, as well as other abdominal organs, are often visible on these scans. Therefore, it is possible that a non-invasive assessment of renal sphingolipid accumulation in FD could be adequately performed with images acquired from research and/or clinical CMR scans. Currently, it is unknown if there are differences in renal native T1 between healthy individuals and FD patients. Therefore, the aim of this study was to evaluate whether cardiac-dedicated research native T1 maps can be used to detect renal sphingolipid accumulation in FD patients, defined as low renal native T1 compared to healthy subjects, and explore native T1 in the spleen, liver, and blood in both FD and healthy subjects.

## 2 Methods

### 2.1 Study groups

In this clinical retrospective cohort study, FD patients who had undergone clinical CMR between 2015 and 2020 were recruited from the outpatient clinic at the Endocrinology Department at Karolinska University Hospital. All FD patients who had undergone a clinically indicated CMR scan, and accepted inclusion for retrospective analysis for research purposes, were included. The diagnosis of FD had been determined by alpha-galactosidase A activity in leukocytes and confirmed by genetic analysis of the *GLA* gene. A population of 38 healthy subjects from 2 different study cohorts [16], [17] without cardiovascular symptoms and contraindications for CMR scans, who had voluntarily undergone 1 research CMR between 2016 and 2022, were included. The inclusion criteria for the healthy subjects were that they had no previous history of cardiovascular symptoms, kidney disease, and/or asthma, had a normal creatinine value, and were current non-smokers. A venous blood sample, used to determine blood hematocrit and creatinine, was collected immediately before the CMR scan for both groups. No specific instructions regarding hydration before the CMR scan were given. The cohort of FD patients included several patients who had undergone multiple CMR scans on a clinical basis, where the images from the most recent scan were included in the study. All participants provided written informed consent and ethical approval was granted from the ethical review board in Stockholm, ethical review numbers: Dnr 2015/2106-31/1, Dnr 2011/1077-31/1, and Swedish Ethical Review Board Authority Dnr 2021-06837-02.

### 2.2 Image acquisition

Short-axis native T1 maps (3–15 slices) were acquired with a 1.5T scanner at Karolinska University Hospital (MAGNETOM Aera, Siemens Healthcare, Erlangen, Germany), using anterior and posterior phased-array surface coils with the participants lying supine. Native T1 maps were acquired with two different single-breath-hold electrocardiographically gated modified Look-Locker inversion recovery (MOLLI) sequences with single-shot balanced steady-state free precession (bSSFP) readout, motion correction, and automatic inline T1 map generation. Complete scanning protocols are available in Appendix A. FD patients and 15 healthy volunteers were acquired with a research MOLLI 5s(3s)3s sampling scheme (works in progess (WIP) sequence 1041B) (in total n = 33, MOLLI_patientcohort and MOLLI_healthycohort2 in Appendix A), and 23 healthy volunteers were acquired with a 5(3)3 sampling scheme (product sequence, MOLLI_healthycohort1 in Appendix A). For the 5s(3s)3s scheme, the number of acquisition/rest heartbeats was calculated to achieve the duration in seconds during scan preparation and rounded down as a deviation in vendor-provided protocols from previously described and published methods, always including a minimum of eight acquisitions. The median acquisition time from first to last image acquisition for MOLLI 5s(3s)3s was 11 s, (min: 8 s, max: 18 s, IQR: 9–12 s), and the median acquisition time for MOLLI 5(3)3 was 10 s (min: 7 s, max: 14 s, IQR: 9–11 s). For both sampling schemes images were acquired in short-axis slices in end-diastole (end of data acquisition, 80%–90% of the R-R interval) [17]. Typical parameters included flip angle (FA) 35°, pixel size 1.4 × 1.9 mm^2^ (reconstructed 1.4 × 1.4 mm^2^), slice thickness 8.0 mm, echo time (TE) 1.19 ms, repetition time (TR) 2.7 ms, matrix size = 144 × 256, field of view (FOV) 270 × 360 mm^2^.

A retrospectively electrocardiogram-gated bSSFP cine imaging covering the entire left ventricle (LV) in short-axis slices, as well as one slice in each of the three long-axis, was acquired to assess LV function. Typical imaging parameters included FA 68°, pixel size 1.9 × 1.4 mm^2^, slice thickness 8.0 mm, TE/TR 1.16/ 36.14 ms, matrix size = 144 × 256, and FOV 270 × 360 mm^2^.

### 2.3 Image analysis

The native T1 maps from FD patients and healthy subjects were analyzed using Segment® software (Medviso AB, Lund, Sweden) with regard to the visibility of kidneys, liver, and spleen. Native T1 values in the kidneys, liver, spleen, heart, and blood were measured by one observer by manually delineating regions of interest (ROI), conservatively placed with a minimum gap of 1 pixel between adjacent structures, in the renal cortex, renal medulla, heart, spleen, blood, and liver. All images and T1 maps were reviewed for artifacts and residual motion misalignment before the analysis process; however, no artifacts and residual motion misalignments were found. Renal cortex, medulla, spleen, and liver ROIs were drawn in the slice where most parenchyma was visible. Endo- and epicardial borders were delineated in all short-axis slices of the myocardium and averaged across all slices. Blood ROIs were placed in the LV blood pool of a midventricular slice. To assess interobserver variability, image analysis of the respective native T1 maps of 15 healthy subjects was performed by one additional independent observer. In addition, image analysis was done one additional time by the first observer of 15 healthy subjects to assess intraobserver variability.

LV volumes, ejection fraction, and myocardial mass were quantified using the software syngo.via (Siemens Healthcare, Erlangen, Germany), with automatic endo- and epicardial contour tracking with manual corrections. Body surface area (BSA) was calculated with the Dubois formula [18]. Volumetric measurements and myocardial mass were indexed to BSA.

### 2.4 Statistical analyses

All data obtained were collected and analyzed in Microsoft Excel® (Microsoft, Redmond, Washington). Parameters with continuous values were tested for normal distribution by Shapiro-Wilk test, and if normally distributed, they were presented as mean ± standard deviation (SD). Non-normally distributed values were presented with median and interquartile range (IQR). Mean values of native T1 in the renal cortex, renal medulla, heart, liver, spleen, and blood were compared between FD patients and healthy subjects using linear and logistic regression models. Linear regression models were used to assess analyses of repeated values for the patients who did repeated examinations. The relationship between renal native T1 and the estimated glomerular filtration rate (eGFR) (calculated using the revised Lund-Malmö equation [19]), and the age of the FD patients and the healthy subjects were analyzed using linear regression models, and data presented as R^2^, respectively. Inter- and intraobserver variabilities were calculated using intraclass correlation (ICC) as a measure of agreement. Inter- and intraobserver disagreements were presented as absolute differences with SD. All p-values <0.05 were considered statistically significant. Statistical analyses were performed using STATA software (version 16.1 Stata Corp., College Station, Texas).

## 3 Results

In total, 18 FD patients and 38 healthy subjects were included for analysis, baseline characteristics are presented in Table 1. The cohort of FD patients and the cohort of healthy subjects were matched on a group basis for mean age (40.8 years in both groups, p = 1.00). The set of healthy subjects included 18 males and 20 females, while the cohort of FD patients included 8 males and 10 females. In total, three patients were on enzyme treatment before the CMR examination, with a mean treatment duration of 4.5 years (range 2–7 years). The kidneys were visible in 36 (95%) of the 38 healthy subjects and in 100% of the FD patients. All native T1 values are presented in Table 2. There was a difference in native T1 between the FD patients and healthy subjects in the heart and renal medulla; however, there were no differences in the renal cortex, spleen, liver, or blood. Ten of the FD patients underwent a follow-up CMR examination after the first examination included for analysis, and six of these patients underwent a third follow-up CMR examination. Notably, the cortex and medullary T1, respectively, did not increase over time (p = 0.51 and p = 0.18, respectively).

**Table 1:** Baseline characteristics of healthy subjects and FD patients.

Characteristic	Healthy subjects (n = 38)	FD patients (n = 18)	p-value
Sex Female, n (%) Male, n (%)	20 (53) 18 (47)	10 (56) 8 (44)	0.84
Age, years Female Male	41 ± 16 42 ± 16 39 ± 15	41 ± 10 41 ± 12 41 ± 8	1.00 0.79 0.78
Weight, kg Female Male	73 ± 12 67 ± 11 79 ± 9	77 ± 17 76 ± 15 76 ± 20	0.37 0.06 0.61
Length, cm Female Male	175 ± 9 168 ± 6 183 ± 22	175 ± 9 171 ± 4 181 ± 12	0.88 0.14 0.52
BSA* Female Male	1.9 ± 0.2 1.8 ± 0.1 2.0 ± 0.1	1.9 ± 0.2 1.9 ± 0.2 2.0 ± 0.3	0.52 0.04 0.48
Creatinine, µmol/L Female Male	72 ± 15 63 ± 13 81 ± 10	74 ± 20 67 ± 13 82 ± 24	0.71 0.59 0.92
eGFR,** mL/min/1,73 m^2^ Female Male	90 ± 15 91 ± 19 88 ± 11	90 ± 17 89 ± 15 90 ± 20	0.95 0.82 0.70
Hematocrit, % Female Male	42 ± 4 40 ± 3 45 ± 3	41 ± 3 42 ± 3 40 ± 2	0.37 0.11 **<0.01**
Heart rate, beats/min Female Male	71 ± 9 70 ± 8 73 ± 11	59 ± 10 62 ± 11 56 ± 6	**<0.01** **0.03** **<0.01**
Cardiac index, L/min/m^2^ Female Male	3.8 ± 1.0 3.3 ± 0.8 4.3 ± 1.0	3.1 ± 0.7 2.7 ± 0.2 3.6 ± 0.8	**0.03** **0.02** 0.12
LVEF, % Female Male	58 ± 5 58 ± 6 59 ± 5	63 ± 5 62 ± 5 64 ± 5	**0.01** 0.12 **0.03**
LVmass, g Female Male	116 ± 28 95 ± 14 139 ± 21	163 ± 67 118 ± 26 213 ± 64	**<0.01** **<0.01** **<0.01**
LVMi, g/m^2^ Female Male	61 ± 10 54 ± 6 69 ± 8	85 ± 32 62 ± 13 110 ± 29	**<0.01** **0.04** **<0.01**
LVEDV, m Female Male	170 ± 42 142 ± 24 202 ± 34	170 ± 45 146 ± 28 196 ± 47	0.94 0.65 0.68
LVEDVi, mL/m^2^ Female Male	90 ± 17 81 ± 13 100 ± 15	88 ± 19 77 ± 16 100 ± 15	0.73 0.56 0.92
LVESV, mL Female Male	71 ± 20 59 ± 10 84 ± 19	63 ± 17 56 ± 10 71 ± 21	0.17 0.42 0.15
LVESVi, mL/m^2^ Female Male	37 ± 8 33 ± 5 41 ± 8	33 ± 7 30 ± 6 36 ± 7	0.06 0.11 0.13

Values presented as mean ± SD (all values were normally distributed). p-Values denote logistic or linear regression as appropriate. Statistically significant p-values are in bold.

*FD* Fabry disease, *BSA* body surface area, *eGFR* estimated glomerular filtration rate, *LVEDV* left ventricular end-diastolic volume, *LVEF* left ventricular ejection fraction, *LVESV* left ventricular end-systolic volume, *LVmass* left ventricular mass, *LVMi* left ventricular mass index, *LVEDVi* left ventricular end-diastolic volume index, *LVESVi* left ventricular end-systolic volume index, *SD* standard deviation.

* BSA was calculated using the formula of DuBois [20]

** eGFR was calculated using the revised Lund-Malmö equation [19]

**Table 2:** Native T1 in FD patients and healthy subjects.

Group	Cortex (ms)	Medulla (ms)	Heart (ms)	Spleen (ms)	Liver (ms)	Blood (ms)
FD patients	1034 ± 88	1617 (1548–1661)	951 ± 79	1143 ± 45	557 (525–592)	1596 (1550–1626)
Healthy subjects	1056 ± 59	1514 ± 81	1006 ± 38	1132 ± 70	557 ± 47	1576 ± 100
p-value	0.29	**<0.01**	**<0.01**	0.54	0.41	0.22

Data are native T1 values in milliseconds (ms) presented as mean ± SD for normally distributed values, for non-normally distributed values, median, and interquartile range (IQR) are presented. p-Values denote linear regression models. Statistically significant p-values are in bold.

*FD* Fabry disease*, SD* standard deviation

### 3.1 Sex differences in native T1

**Fig. 1:**
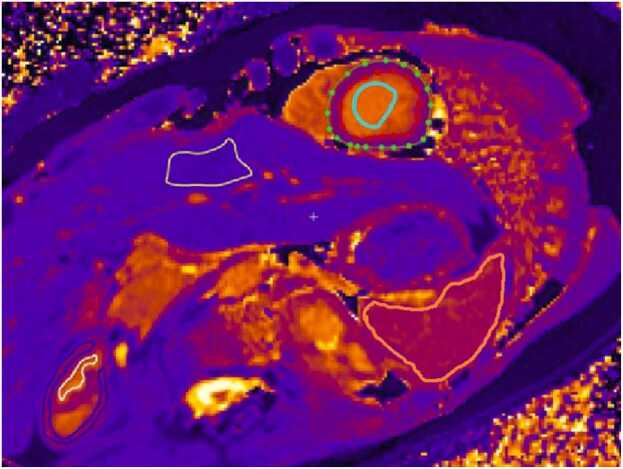
Regions of interest (ROI). Representative example of ROI of native T1 for renal cortex, renal medulla, spleen, liver, and heart. Blue ROI = renal cortex, white ROI = renal medulla, orange ROI = spleen, yellow ROI = liver, green/red dotted ROI = heart, and light blue ROI = blood

**Fig. 2:**
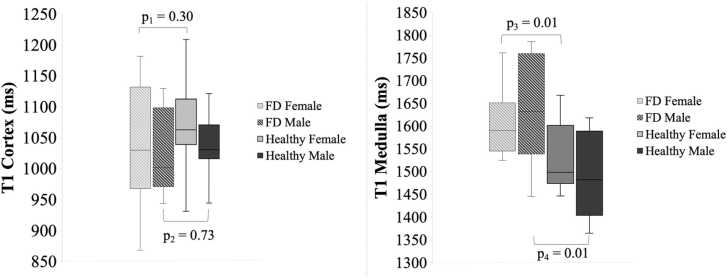
Sex differences in native T1 in the renal cortex and medulla. Native T1 in the renal cortex and renal medulla in females and males compared between healthy subjects and Fabry disease (FD) patients. Sex-based comparison between FD patients and healthy subjects is presented with p-values; p_1_ = comparison of T1 cortex between FD females and healthy females, p_2_ = comparison of T1 cortex between FD males and healthy males, p_3_ = comparison of T1 medulla between FD females and healthy females, p_4_ = comparison of T1 medulla between FD males and healthy males

There were native T1 differences between FD patients and healthy subjects in the heart and renal medulla in both men and women and a difference in the spleen only in males (Fig. 1). There were no differences in native T1 in the renal cortex, liver, and blood for both males and females in analyses of the whole study group (Figs. 1 and 2).

### 3.2 Relationships between native T1, renal function, and age

There was no correlation between the native T1 in the renal cortex and medulla, with eGFR and age, respectively, among the healthy subjects (Appendix B) nor FD patients (Appendix C). In the 75th quantile of patients with the highest eGFR (>101 mL/min/1,73 m^2^), FD patients had lower native T1 in the renal cortex compared to healthy volunteers (FD patients vs healthy volunteers; 998 vs 1071 ms, p = 0.03); however, no difference was seen in the 25th quantile of patients with the lowest eGFR (<80 mL/min/1,73 m^2^). Further, no differences in native T1 of the medulla, spleen, or liver were seen between healthy subjects and FD patients in the 25th quantile with the lowest eGFR, nor in the 75th quantile with the highest eGFR. When stratified for sex, there was no correlation between the native T1 in the renal cortex, with eGFR and age, respectively, in male healthy subjects (eGFR: R^2^ < 0.01, p = 0.84; age: R^2^ ≤ 0.01, p = 0.84) nor in male FD patients (eGFR: R^2^ = 0.06, p = 0.61; age R^2^ < 0.01, p = 0.97).

There were no correlations between hematocrit and native T1 of the renal medulla in FD patients (R^2^ < 0.01, p = 0.81) or in healthy subjects (R^2^ = 0.04, p = 0.24). Further, there were no correlations between hematocrit and native T1 of the renal medulla in female (R^2^ = 0.03, p = 0.68) or male (R^2^ = 0.16, p = 0.38) FD patients, nor in female (R^2^ < 0.01, p = 0.93) or male (R^2^ = 0.03, p = 0.55) healthy subjects. Also, there were no correlations between blood native T1 and native T1 of the renal medulla in FD patients (R^2^ < 0.01, p = 0.82) or in healthy subjects (R^2^ = 0.29, p = 0.09). Additionally, there were no correlations between blood native T1 and native T1 of the renal medulla in female (R^2^ = 0.08, p = 0.44) or male (R^2^ = 0.02, p = 0.75) FD patients, nor in female (R^2^ = 0.02, p = 0.62) or male (R^2^ = 0.17, p = 0.11) healthy subjects.

### 3.3 Intra- and interobserver analysis

Intra- and interobserver measures of agreement and disagreement are presented in Table 3.

**Table 3:** Inter- and intraobserver agreement and disagreement.

	Interobserver		Intraobserver	
	ICC	Difference (95%CI)	ICC	Difference (95%CI)
T1 cortex	0.85, p < 0.01	4.7 (0.5–65.9)	0.97, p < 0.01	10.6 (2.3–30.7)
T1 medulla	0.75, p < 0.01	133.0 (97.3–224.2)	0.95, p < 0.01	16.5 (2.8–88.0)
T1 heart	0.95, p < 0.01	25.5 (8.2–88.2)	0.95, p < 0.01	13.1 (0.5–30.3)
T1 spleen	0.77, p < 0.01	60.4 (19.8–196.9)	0.99, p < 0.01	1.5 (0.1–12.4)
T1 liver	0.93, p < 0.01	95.0 (33.1–102.8)	0.97, p < 0.01	8.1 (0.6–44.6)

Each subject was assessed by two raters. Data are presented as intraclass correlation coefficients (ICCs) that were obtained using two-way mixed-effects models as a measure of agreement. Disagreement is presented as absolute median differences with 95% confidence intervals (CI)

## 4 Discussion

In this retrospective study, we show that there are no differences in native T1 in the renal cortex or abdominal organs between FD patients and healthy subjects in cardiac-dedicated scans, despite measurable cardiac sphingolipid accumulation. Native T1 in all measured organs in healthy subjects was in line with previously published values [18], [21], [22], [23], [24], thus supporting that native T1 can be measured in abdominal organs in research cardiac CMR scans. However, no difference in renal cortex native T1 was observed between healthy subjects and FD patients.

### 4.1 Differences in the renal medulla between FD patients and healthy subjects

One of the more surprising results of this study was that the native T1 in the renal medulla was higher in FD patients than in the healthy subjects. Since there were no differences in the native T1 in the blood of the healthy subjects and the FD patients, this difference cannot totally be explained by image acquisition-based factors, and other physiological factors are therefore likely to contribute. One may hypothesize that the differences may be due to the hyperfiltration state that the kidneys undergo before filtration starts to decline in FD patients. Previous studies have theorized that the wide range of native T1 in the renal medulla among both normal and diseased kidneys may be due to differences in perfusion, different cellular processes, or interstitial changes in the kidneys [15]. However, how factors such as interstitial changes have an impact on native T1 in the medulla is yet to be fully understood [15].

### 4.2 No difference in abdominal native T1 between FD patients and healthy subjects

Physiologically, the FD patients in this study had cardiac involvement as their left ventricular mass clearly shows hypertrophied hearts and lower myocardial native T1; however, there was no difference in kidney function between FD patients and healthy subjects. Among the patients with the highest eGFR, FD patients had lower native T1 in the renal cortex compared to healthy volunteers; however, no difference was seen among the patients with the lowest eGFR. This may suggest that renal sphingolipid accumulation can be identified very early on, long before renal function declines. Further, the off-resonance effect rendering longer T1 in the presence of fat could possibly contribute to the relatively long T1 values in the renal parenchyma. Given the many physiological factors affecting the renal parenchyma, it may be very complex to identify renal sphingolipid accumulation. An aspect to take into consideration is that the sphingolipid accumulation is heterogenic; however, it most likely increases over time, and our relatively young patient cohort may not have enough sphingolipid accumulation to differ them from healthy objects, when measuring native T1 in the kidneys, despite that the patients have cardiac involvement. Another aspect of native T1 in the kidney in a cardiac-dedicated scan is partial volume effects, which potentially could lead to a dilution of native T1 of the renal cortex, that could be mitigated by kidney-dedicated native T1 mapping.

However, there was a difference in spleen native T1 values between male FD patients and healthy males. Sphingolipid accumulation in the spleen is, to our knowledge, not a known manifestation of FD. The difference in native T1 values in the spleen could rather be explained by an effect of different spleen perfusion, age, or other image-based or physiological factors beyond the scope of the current study.

### 4.3 Impact of eGFR and age on native T1 of the kidney

There were no correlations between native T1 in the kidney and eGFR or age, respectively.

**Fig. 3:**
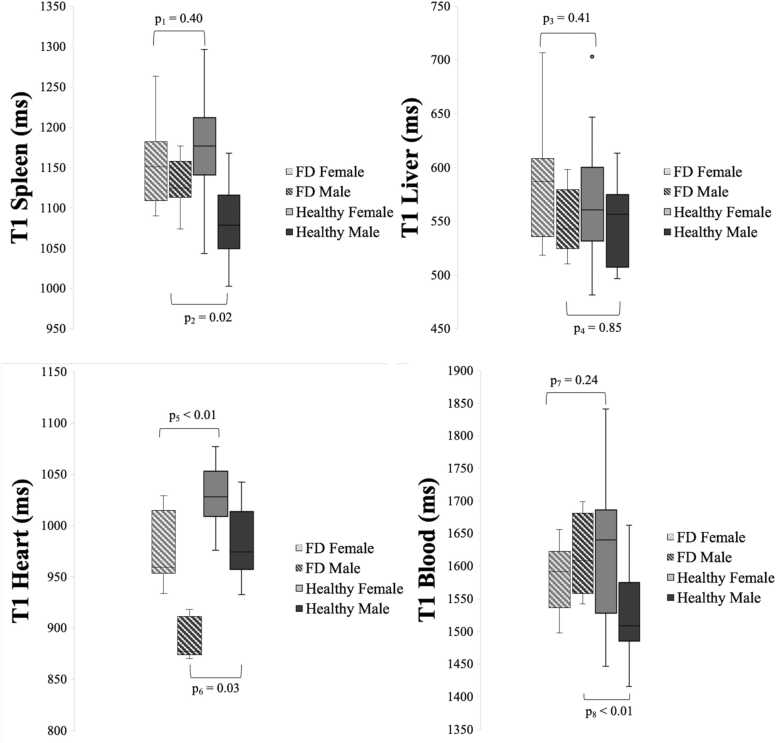
Sex differences in native T1 in the heart, spleen, liver, and blood. Native T1 in the heart, spleen, liver, and blood in females and males compared between healthy subjects and FD patients. Sex-based comparison between FD patients and healthy subjects is presented with p-values; p_1_ = comparison of T1 spleen between FD females and healthy females, p_2_ = comparison of T1 spleen between FD males and healthy males, p_3_ = comparison of T1 liver between FD females and healthy females, p_4_ = comparison of T1 liver between FD males and healthy males, p_5_ = comparison of T1 heart between FD males and healthy males, p_6_ = comparison of T1 heart between FD males and healthy males, p_7_ = comparison of T1 blood between FD males and healthy males, p_8_ = comparison of T1 blood between FD males and healthy males. *FD* Fabry disease

The normal aging process in the kidney causes a slight loss of corticomedullary differentiation, as the normal aging kidney undergoes structural deterioration and progressive decline in renal function, leading to cortical atrophy [25]. A loss of corticomedullary differentiation is often primarily attributed to increased T1 relaxation times in the renal cortex [15], hence an increasing native T1 in the kidneys can be seen with increasing age. Loss of corticomedullary differentiation has been observed in renal insufficiency, secondary to a variety of conditions, including glomerulonephritis, acute tubular necrosis, end-stage chronic renal failure [15] as well as in FD [26]. The data of this study rather show the opposite, a trend toward that native T1 increases with decreasing eGFR in the renal cortex of the FD patients (R^2^ = 0.16, p = 0.14, Fig. 3). This may reflect a development of atrophy in the kidneys due to FD, rather than a considerable cortical sphingolipid accumulation. This could support our hypothesis showing no increase of native T1 in renal cortex among FD patients over time.

## 5 Limitations

One of the strengths of this study of native T1 in the kidney and abdominal organs in FD patients is the high intra- and interobserver agreement, thus supporting the notion that native T1 can be measured in abdominal organs in cardiac-dedicated research native T1 maps. This study has three major limitations: the small FD cohort, the different scanning protocols due to retrospective analysis of different cohorts, and image-based factors such as partial volume and off-resonance effects. The obvious limitation is the low number of FD patients (18 in total, 8 of these being males). However, there were differences in cardiac native T1 between FD patients and healthy volunteers, but no difference in renal native T1. To better understand why there were no differences in native T1 from the kidneys, it would have been valuable to validate the results with an independent test for renal sphingolipid accumulation in patients and subjects, such as renal biopsies could have been used [13], [14].

Another limitation of the study was that two different MOLLI sampling schemes were used in this retrospective analysis, (5s(3s)3s) and (5(3)3), due to routine at different time points. Furthermore, the vendor-provided seconds-based protocol deviated from previously described and published methods. One of the major issues with MOLLI acquisition is the heart rate dependency, especially for long T1 values, where higher heart rates cause imperfect inversions if the magnetization has not fully recovered, leading to underestimation of native T1. This issue increases with increased heart rate [27], where the accuracy degrades at higher heart rates; however, precision is still maintained. In our subjects, there was no difference in heart rate across the different sampling protocols in the healthy volunteers (heart rate (HR) MOLLI 5(3)3 vs MOLLI 5s(3s)3s; 72 vs 70 bpm, p = 0.62), and only one subject had heart rate >90/min (i.e. 101/min), so even though we expect good precision given the similar heart rates, the accuracy of some of the native T1 values may be worse with the MOLLI 5(3)3 scheme. A possible degradation of accuracy would affect both heart and kidneys similarly; however, this possible underestimation is not evident in the cardiac native T1 values which are lower in the FD patients. Other image-based limitations include partial volume effects, as the slice thickness is 8 mm, the renal cortex is around 6–7 mm, and the kidneys are suboptimally positioned in heart-dedicated images. Acquired images cannot be changed; however, we tried to mitigate partial volume effects by careful delineation of the different structures, with ROIs drawn at least one pixel’s distance between different structures. Kidney-dedicated imaging may mitigate partial volume effects even further. Also, when measuring peripheral organs in a cardiac-dedicated scan, one potential concern is off-resonance effects [28]. Off-resonance effects are clinically often mitigated with a local shim box, allowing for a uniform field around the heart. However, the study was performed at 1.5T where off-resonance effects are reduced, and no visible bSSFP banding artifacts were present near the organs quantified.

The findings suggest that men with FD had a lower hematocrit and higher blood T1. The relationship between lower hematocrit and higher blood T1 is known and described in the literature [29]. In a study of 345 patients with FD, anemia was present in 47% of males and 20% of females, indicating anemia is more common among male patients with FD [30]. This might be a contributing factor to the lower hematocrit among males with FD in our study. It would be valuable to analyze further sex differences in FD patients in future studies. Another limitation is that we did not perform corrections for multiple comparisons as we considered the comparisons to be relatively few, rendering a possibility of spurious findings.

Taking into account that this study was retrospective and not originally focused on the kidneys imaging-wise rendering that the slice orientation may not be ideal, these results need to be validated in larger cohorts, with kidney-focused images, and kidney-optimized native T1 mapping may increase diagnostic sensitivity as this mitigates partial volume effects and off-resonance effects.

## 6 Conclusions

Compared to healthy subjects, patients with FD and cardiac involvement had no differences in native T1 of the renal cortex. FD patients had higher native T1 in the renal medulla, which was not totally explained by differences in blood native T1. The findings suggest that sphingolipid accumulation in the renal cortex in FD patients could not be detected with the cardiac-dedicated research native T1 maps used in this study, relaying on opportunistic images of the kidney. However, the higher native T1 in the renal medulla may reflect the hypothesis of a hyperfiltration state in the development of renal failure. More research with kidney-focused native T1 mapping in FD is needed to corroborate these results.

## Funding

The research was funded in part by the 10.13039/501100004359Swedish Research Council, Swedish Heart and Lung Foundation, the Region of Stockholm and Karolinska Institutet.

## Author contributions

A.D. participated in the design of the study, performed statistical analysis, contributed to the interpretation of data, and drafted the manuscript. F.K. participated in study design, performed image analysis, contributed to the interpretation of data, and helped to draft the manuscript. R.T. participated in study design, image acquisition, and revised the manuscript. K.C. provided the research sequences and technical expertise, and revised the manuscript. M.O. participated in study design, patient recruitment, interpretation of data, and revised the manuscript. J.N. conceived the study and its design, performed image acquisition and image analysis, contributed to the interpretation of data, and helped to draft the manuscript. All authors read and approved the final manuscript.

## Ethics approval and consent

All subjects provided written informed consent and ethical approval was granted from the ethical review board in Stockholm, ethical review numbers: Dnr 2015/2106-31/1, Dnr 2011/1077-31/1, and Swedish Ethical Review Board Authority Dnr 2021-06837-02.

## Consent for publication

Written informed consent was obtained from patients for publication of their details on a group level and anonymized images in this manuscript. The consent form is held in the patients’ clinical notes and is available for review by the Editor-in-Chief.

## Declaration of competing interests

The authors declare the following financial interests/personal relationships which may be considered as potential competing interests: Jannike Nickander report financial support was provided by Swedish Research Council, Swedish Heart and Lung Foundation, and Region of Stockholm. Kelvin Chow reports a relationship with Siemens Healthcare that includes employment. Jannike Nickander reports a relationship with Sanofi Genzyme Sweden that includes speaking and lecture fees. Mikael Oscarson reports a relationship with Sanofi Genzyme Sweden that includes speaking and lecture fees. The other authors declare that they have no known competing financial interests or personal relationships that could have appeared to influence the work reported in this paper.

## Figures and Tables

**Figure:**
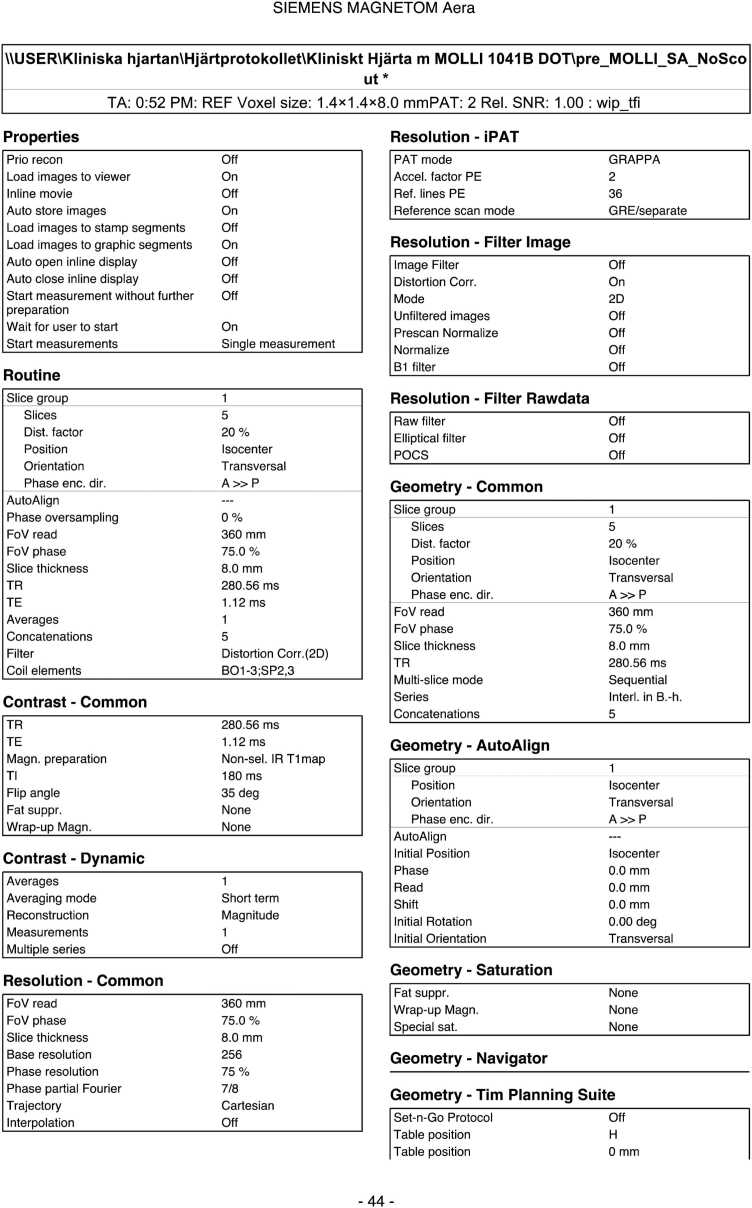


**Figure:**
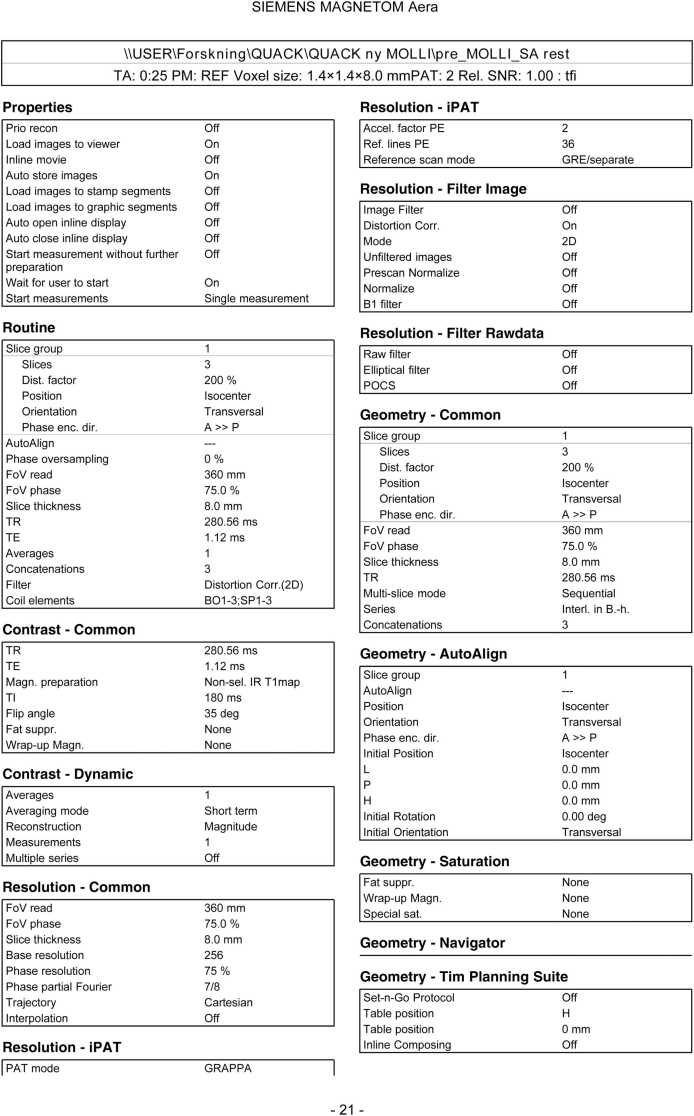


**Figure:**
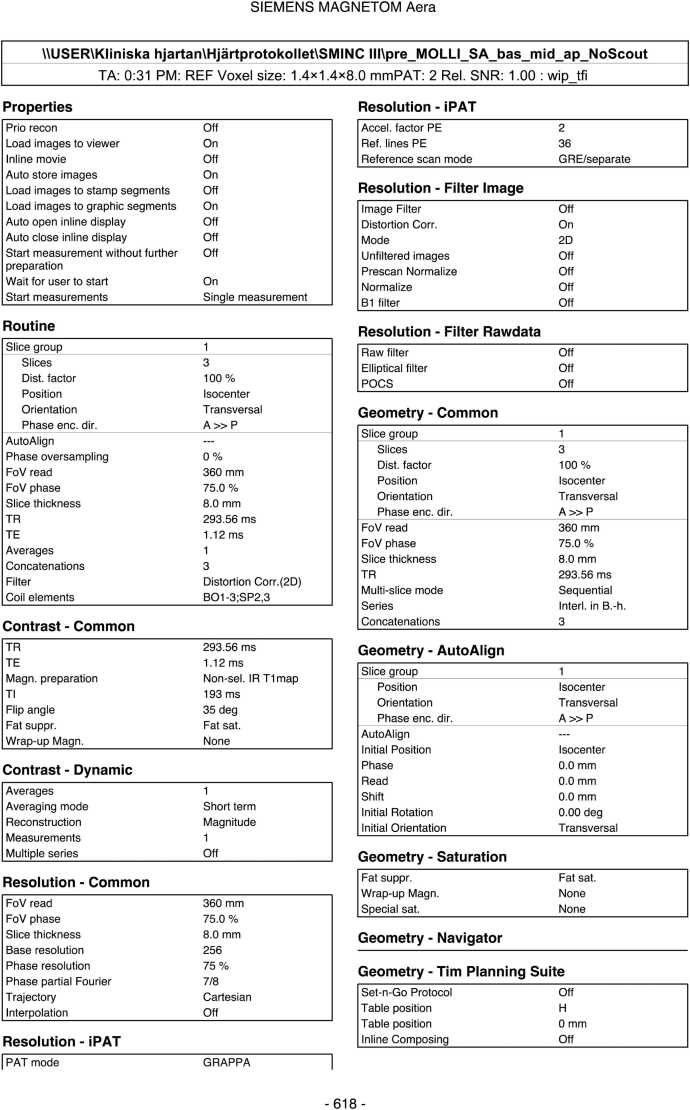


**Figure:**
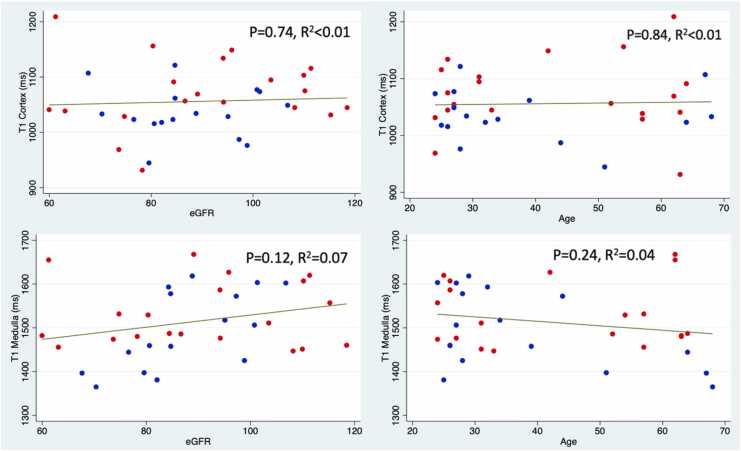


**Figure:**
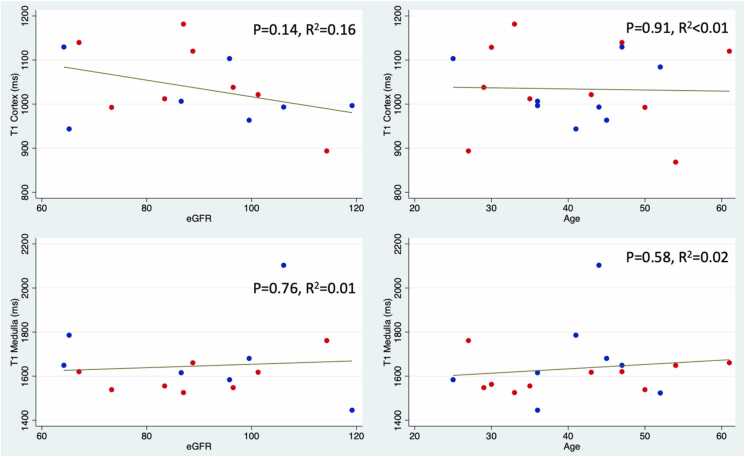


## Data Availability

The data that support the findings of this study are available from the corresponding author upon reasonable request.

## Appendix A Printout of scan protocols

.

Notes: “wip_tfi” is a non-product research sequence and “tfi” is a product sequence.

## Appendix B Relationship between renal native T1, age, and renal function in healthy subjects

.

Native T1 in the cortex and medulla together with eGFR and age. Male patients in blue and female in red.

## Appendix C Relationship between renal native T1, age, and renal function in FD patients

.

Native T1 in the cortex and medulla together with eGFR and age. Male patients in blue and female in red.
